# Structure of the xenotropic murine leukaemia virus-related virus matrix protein

**DOI:** 10.1186/1742-4690-8-S1-A227

**Published:** 2011-06-06

**Authors:** Michal Doležal, Iva Pichová, Tomáš Ruml, Richard Hrabal, Michaela Rumlová

**Affiliations:** 1 Institute of Organic Chemistry and Biochemistry, IOCB Research Centre and Gilead Sciences, Academy of Sciences of the Czech Republic, Prague, 166 10, Czech Republic; 2 Department of Biochemistry and Microbiology, Institute of Chemical Technology Prague, Prague, 166 28, Czech Republic; 3 Laboratory of NMR spectroscopy, Institute of Chemical Technology Prague, Prague, 166 28, Czech Republic

## 

We present the preparation of the xenotropic murine leukaemia virus-related virus matrix protein (XMRV-MA) and its structure determined by NMR spectroscopy.

The DNA fragment encoding XMRV-MA was obtained from prostate tumour cell cDNA (Rv1 cell line) by PCR and inserted into a pET-22b plasmid. Non-myristoylated, uniformly 13C- and 15N-labeled XMRV-MA, fused with histidine tag, was produced in E. coli BL21 (DE3) cells. The protein was purified by immobilized metal affinity chromatography (NiNTA-agarose) and size-exclusion chromatography (Sephadex 75), and then concentrated to 5 mg/ml.

All NMR data were collected at 298 K on a 600 MHz Bruker Avance III spectrometer equipped with a cryogenic triple-resonance probe and analyzed with CcpNmr Analysis. Back-bone and side-chain resonances were assigned using standard NMR experiments and structural constraints were obtained from 13C- and 15N-edited NOESY experiments. Structures were calculated with ARIA.

Although the protein sequence of the XMRV-MA is very similar to that of the murine leukaemia virus matrix protein (MLV-MA), it varies in several amino acid residues. We compared the structures of the XMRV-MA and MLV-MA and found that those changes are localized in a few domains, mostly on the surface of the protein.

